# Editorial: Updates on Osteoimmunology: What's New on the Crosstalk Between Bone and Immune Cells

**DOI:** 10.3389/fendo.2020.00074

**Published:** 2020-02-20

**Authors:** Giacomina Brunetti, Patrizia D'Amelio, Giorgio Mori, Maria Felicia Faienza

**Affiliations:** ^1^Section of Human Anatomy and Histology, Department of Basic and Medical Sciences, Neurosciences and Sense Organs, University of Bari, Bari, Italy; ^2^Gerontology Section, Department of Medical Sciences, University of Torino, Turin, Italy; ^3^Centre Hospitalier Universitaire Vaudois (CHUV), Lausanne, Switzerland; ^4^Department of Clinical and Experimental Medicine, University of Foggia, Foggia, Italy; ^5^Paediatric Unit, Department of Biomedical Science and Human Oncology, University of Bari, Bari, Italy

**Keywords:** osteoimmunology, immune cell, osteoclast, osteoblast, osteocyte

Osteoimmunology is an important field of bone research, it deepens the crosstalk between bone, and immune cells in both physiological and pathological conditions (1). The relative mechanisms were reported in the papers of this special issue, grouped into different categories: general mechanisms, pathological conditions, and potential therapeutics.

## Osteoimmunology General Mechanisms

Ponzetti and Rucci reviewed in detail this topic. They reported that many cytokines that are considered immune-related, such as Interleukins, Tumor Necrosis Factor (TNF)-α, and Receptor-Activator of Nuclear factor Kappa B Ligand (RANKL), have all been demonstrated to be critical in osteoblast and osteoclast biology. On the other hand, bone cells, crucial for bone remodeling, actually regulate immune cells, and thus the endosteal niche. Both osteoclasts and osteoblasts take part to this niche, either by supporting engraftment, or mobilization of Hematopoietic Stem Cells (HSCs).

Interestingly, Lombardi et al. explored the effect of physical activity on bone metabolism, taking into account not only the direct role of biomechanical load, but also focusing on the role of immunomodulation. Although the majority of the studies point to the inflammation-mediated effects of physical activity, it has been shown that it regulates immune function through the inflammasomes, and through the secretion of myokines and adipokines. Although it is known that regular physical activity is effective in improving and maintaining bone mass, there is no consensus on the best kind of exercise to prescribe.

Additionally, Xiao and Xiao described autophagy involvement in osteoimmunology. Autophagy is a cytologic essential event for dysfunctional organelles degradation thus involved in cell survival; the process is also responsible for immune cell polarization (2). Bone cells use autophagy for their main functions, in particular osteoclast differentiation, polarization and adhesion to bone matrix depend on autophagy (3); osteoblasts through their phagosomes secrete calcium phosphate crystals in extracellular matrix supporting osteoid matrix mineralization (4). Differently, autophagy inhibits osteocytes oxidative stress consequences, neutralizing damaged mitochondria that could induce apoptosis (5). The authors concluded that autophagy, driving the inflammatory process in the immune cells, have important consequences on bone tissue not only in physiological remodeling but also in the diseases.

Interestingly, Fischer et al. revised the role of Foxp3+ regulatory T cells, a particular subset of CD4+ T cells, in bone and hematopoietic homeostasis (6, 7). Foxp3+ Treg cells play a crucial role in preserving immune homeostasis, but may also control regenerative and metabolic processes, osteoclast activity, and differentiation of HSCs. Foxp3+ Treg cells affect lympho-haematopoiesis through indirect mechanisms, i.e., by acting on osteoclast differentiation and activity, which determines changes in the niche size.

In the review by Metzger and Narayanan the authors synthetized the role of osteocytes in immune mediated bone pathological events (8). Aging, changes the canalicular communication between osteocytes impairing their communication features, moreover, in old mice, osteocytes increase the production of inflammatory cytokines, increasing bone resorption. Pro-inflammatory signals can directly determine osteocytes apoptosis affecting bone turnover, or can stimulate osteocytes in the production of anti-osteoblastic or pro-osteoclastic cytokines stimulating bone loss.

## Osteoimmunology and Pathological Conditions

Osteoimmunology key mechanisms have been also highlighted in different pathological conditions (9–12), and in this special issue on obesity, osteopetrosis, osteolytic solid tumors, and multiple myeloma.

Childhood obesity is one of the main health problems worldwide. The excess of adipose tissue leads to inflammation, oxidative stress, apoptosis, and endothelial dysfunctions (13). Severe comorbidities are associated with obesity, most of them developing already in childhood (14). Among these, low bone mineral density, osteoporosis, and fracture risk have been observed in subjects with various degree of obesity. Faienza et al., reviewed the mechanisms involved in obesity-related bone fragility. The authors reported that the accumulation of adipose tissue positively regulates the function of osteoclasts, the bone reabsorbing cells, by up-regulating the production of several cytokines as RANKL, LIGHT, TRAIL, TNFα, and Monocyte Chemotactic Protein-1 (MCP1). In addition, in obese subjects the osteoblastogenesis is inhibited, thereby the result is an enhanced bone resorption.

Osteopetrosis is a rare bone disease characterized by increased bone mass due to defects in osteoclast function or formation (15). Penna et al. deal with this interesting topic reviewing the clinical and genetic heterogeneity of the disease and underscoring the importance of the research in order to develop further innovative treatment for this rare and often lethal disease. Treatment of these patients if often limited by the presence of neurodegeneration, however hematopoietic stem cell transplantation can be successfully applied in several patients, this treatment is effective only during the first months of life, hence a precocious molecular diagnosis is fundamental.

The research article of Zhang et al. reported an analysis on the single-nucleotide polymorphisms (SNPs) and expression of NLRP3 inflammasome related genes, NF-κB, NLRP3, IL-1β, IL-18, Caspase-1, and ASC in 308 acute lymphoblastic leukemia (ALL) patients and 300 healthy controls. Inflammasomes are large cytosolic multiprotein complexes that activate the caspase-1-mediated innate immune responses. The results of this study demonstrated an involvement of NLRP3 inflammasome-related SNPs, especially NF-κB-94ins/del ATTG and CARD8 (rs2043211) genotype, in ALL. Thus, this genetic variant of NLRP3 inflammasome could be a new biomarker and potential targets for ALL. Furthermore, IL-1β (rs16944) and IL-18 (rs1946518) polymorphism seem to predict ALL prognosis. Notably, ALL patients developed osteonecrosis (16), thus is fundamental the identification of new markers as well as therapeutic targets.

Bone is a preferred metastatic site for several cancer and cancer cells may remain dormant for years within bone before growing, thanks to their ability to overcome the immune system (17). Roato and Vitale deal with the interesting and under-investigated topic of bone metastatic niche, the relation with immune system and the role of Natural Killer (NK) cells in the control of bone metastases growth. They suggest that the stimulation of NK cells activity by the use of cytokine combinations and blocking of their checkpoint receptors can be crucial in increasing the opportunity for new therapeutic options in the treatment of bone metastatic disease.

Harmer et al. highlighted the role of interleukin 6 (IL-6) in multiple myeloma, a hematological malignancy of the plasma cells (18). About 80% of patients display the osteolytic bone disease at diagnosis, and different mechanisms have been demonstrated to orchestrate the negative process, such as IL-6 (18). It is an inflammatory cytokine that affects osteoclast differentiation and plasma cell proliferation. In multiple myeloma, IL-6 is responsible of bone homing, osteolytic process, progression as well as drug resistance. The review also reported current and potential therapeutic approaches against IL-6.

## Osteoimmunology and Potential Therapeutics

In the research paper by Massaccesi et al. the authors demonstrated Vitamin E effects on ultrahigh molecular weight polyethylene (UHMWPE). UHMWPE are currently used for joint prothesis, they determine the reduction of wear particles formation with a consequent decrease of free radicals' generation. Bone remodeling activity in sites close to UHMWPE prosthetic devices, can be affected by oxidative stress. Thus, oxidative stress can induce either osteoclast differentiation or osteoblast apoptosis leading to bone resorption. Vitamin E, being a powerful antioxidant molecule, could be proposed in joint prosthetic devices to decrease prothesis wear and at the same time inhibit the influences of the oxidation-induced osteolysis promoting osteoblast survival and osteoblasts apoptosis. The authors concluded that Vitamin E-Stabilization on UHMWPE could be used to increase the life of joint prothesis.

Kishikawa et al. studied the role of Docosahexaenoic acid (DHA) in inflammation-induced osteoclast formation. DHA, an n-3 fatty acid, is an important structural component of the cell membrane, regulates osteoclast differentiation, and activity and has a potent anti-inflammatory effect through G protein-coupled receptor 120 (GPR120), a functional receptor for n-3 fatty acids (19). Kishikawa et al. displayed that both RANKL- and TNF-α-induced osteoclastogenesis was inhibited by DHA. The authors also reported that the number of osteoclasts, bone resorption pits and the level of resorption marker were significantly lower in LPS+DHA-co-administered mice than LPS-administered mice. However, this DHA-induced inhibition was not found in LPS-, DHA-, and selective GPR120 antagonist AH7614-co-administered mice. Thus, the authors concluded that DHA inhibits LPS-induced osteoclast formation and activity *in vivo* via GPR120 by inhibiting LPS-induced TNF-α secretion by macrophages together with the direct inhibition of osteoclastogenesis.

Nicolin et al. focused on the current knowledge on the role of plant-derived polyphenols in suppressing osteoclast differentiation and bone resorption. Polyphenols are natural molecules derived from plants isolated and characterized in the fruits, and vegetables (20). Some of these bioactive compounds have bone anabolic action, by inhibiting bone resorption. Probably, polyphenols could affect bone metabolism through impairment of cytokines which are involved in promoting osteoclast differentiation and resorption. Furthermore, these compounds have antioxidant characteristics, since they can act as scavengers of reactive oxygen species (ROS). However, further studies are needed on the application of these compounds as therapeutic alternative to current therapies (21) in bone diseases.

In conclusion, all the articles dispensed an overview of the mechanisms regulating bone immune cell crosstalk in physiological and pathological conditions, thus identifying possible alternative therapeutic targets.

## Author Contributions

All authors listed have made a substantial, direct and intellectual contribution to the work, and approved it for publication.

### Conflict of Interest

The authors declare that the research was conducted in the absence of any commercial or financial relationships that could be construed as a potential conflict of interest.
